# A review on lipid and polymeric nano-based 17-β-estradiol delivery systems: advances and challenges

**DOI:** 10.3389/jpps.2024.13633

**Published:** 2024-11-15

**Authors:** Mayara Munhoz de Assis Ramos, Fernanda Yamamoto Ricardo-da-Silva, Luiza de Oliveira Macedo, Cristiano Jesus Correia, Luiz Felipe Pinho Moreira, Raimar Löbenberg, Ana Cristina Breithaupt-Faloppa, Nadia Bou-Chacra

**Affiliations:** ^1^ Laboratório de Cirurgia Cardiovascular e Fisiopatologia da Circulação (LIM-11), Instituto do Coração (INCOR), Hospital das Clínicas da Faculdade de Medicina da Universidade de São Paulo, São Paulo, Brazil; ^2^ Departamento de Farmácia, Faculdade de Ciências Farmacêuticas da Universidade de São Paulo, São Paulo, Brazil; ^3^ Division of Pharmaceutical Sciences, Faculty of Pharmacy and Pharmaceutical Sciences, Katz Group-Rexall Centre for Pharmacy and Health Research, University of Alberta, Edmonton, AB, Canada

**Keywords:** nanoparticle, lipid, polymeric, estradiol, treatment

## Abstract

17β-estradiol (E2) is an endogenous steroid hormone pivotal for the development of female secondary sexual characteristics and the maintenance of the female reproductive system. Its roles extend beyond these physiological functions, as E2 is employed in hormone replacement therapy to alleviate symptoms associated with menopause. Furthermore, E2 exhibits therapeutic potential in the management of osteoporosis, breast cancer, and various neurological and cardiovascular conditions, partly due to its anti-inflammatory effects via modulation of the MAPK/NFκB signaling pathway. Notwithstanding, the hydrophobic nature of E2 significantly hinders the formulation of efficacious delivery systems for its clinical deployment. Recent advances have highlighted nano-based delivery systems for E2 as a promising solution to this solubility challenge. This review critically examines contemporary nano-delivery strategies for E2, particularly emphasizing lipid and polymeric nanoparticle-based systems. These nanostructures are designed to enhance stability, biocompatibility, controlled release, and targeted delivery of E2, yet the selectivity of E2 delivery for therapeutic purposes remains an ongoing challenge. The novelty of this review lies in its focus on the advances in nano-based E2 delivery systems over the past decade, a topic not extensively covered in prior literature. We present a comprehensive analysis of the encapsulation of E2 within polymeric and lipid nanoparticles, underscoring the untapped potential of these strategies. This review identifies a significant research gap, advocating for intensified experimental investigations that could pave the way for the translation of nano-based E2 therapies from bench to bedside.

## Graphical Abstract

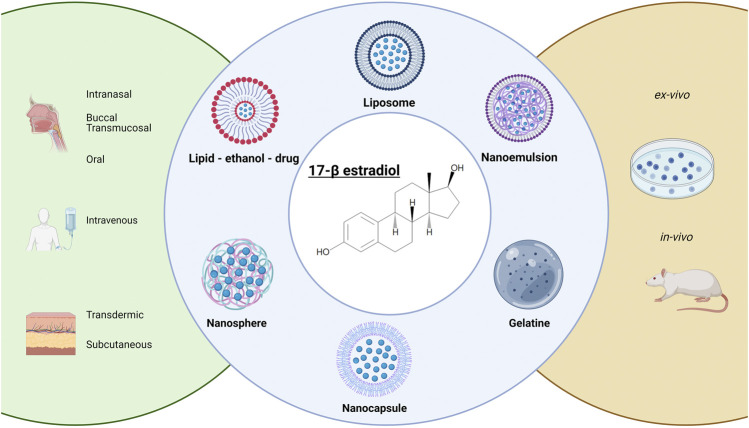

## Introduction

The hormone 17β-estradiol (E2), an endogenous variant of estradiol, is emerging as a promising therapeutic agent for various inflammatory conditions, and it can be administered as a monotherapy or as an adjunct to other treatments. The therapeutic utility of E2 is underscored by its potential to induce fewer adverse effects and drug interactions when compared to its synthetic counterparts. Empirical evidence, such as the investigation conducted by Zhu et al. (2022), corroborates the anti-inflammatory efficacy of E2, particularly in the treatment of diseases like hepatocellular carcinoma, where it modulates the MAPK/NF-kB signaling pathway [1]. Additionally, E2 exhibits a therapeutic role in the management of tendon degeneration by mitigating inflammation and regulating apoptotic processes in tissue [2]. Nonetheless, the lipophilic nature of E2 poses substantial challenges in developing an effective delivery system [3]. Nanotechnology-based drug delivery systems present a solution to this challenge by increasing the bioavailability of E2. These nanostructured systems offer advantages such as targeted drug delivery, controlled release, and enhanced bioavailability, attributed to their prolonged circulation time in comparison to non-encapsulated drugs [4]. The precision targeting capability of nanocarriers not only directs the drug to the specific site of disease but also safeguards the drug from premature metabolic breakdown. Furthermore, the enhanced surface area-to-volume ratio of nanocarriers allows for dosage reduction, thereby improving the safety profile of the drug. The selection of an appropriate nanocarrier, be it polymeric or lipid-based, is determined by the drug’s physicochemical properties and the intended therapeutic outcome. For instance, the U.S. Food and Drug Administration (FDA) has sanctioned the use of Estrasorb™, a nanotechnology-based E2 delivery system, for the alleviation of menopausal symptoms [5]. Clinical studies have validated the safety and efficacy of nanotechnology-amplified hormone replacement therapies, which typically combine estradiol with hormones such as progesterone, in treating menopausal symptoms and reestablishing hormonal equilibrium [6, 7].

Our study reviews recent advancements in the development of polymeric and lipid nanostructured E2 over the past decade, highlighting its therapeutic potential. We examine formulation components, preparation methods, and their *in vivo*/*in vitro* performance, focusing on key factors for advancing the next-generation of these nanoparticles. This review aims to guide strategic decision-making for the future clinical application of nanobased E2 formulations.

## Estradiol (E2): mechanism of action and properties

Cholesterol serves as the foundational substrate for the synthesis of steroid hormones; it undergoes metabolism by a cascade of enzymatic reactions, culminating in the production of the three principal forms of estrogen found in humans: E2, estriol, and estrone [8]. E2 is particularly significant during a woman’s reproductive years, where it is the most abundant estrogen in serum and the most active in terms of estrogenic effects. Due to its high potency and therapeutic effectiveness, E2 is the primary focus for research into estrogen-based therapy. Within endothelial cells, E2 plays a pivotal role in cellular growth, differentiation, and function [9].

E2 exerts its biological effects through interaction with estrogen receptors (ERs), which are categorized into α and β subtypes predominantly located in the cell nucleus and cytoplasm, as well as the G protein-coupled estrogen receptor (GPER, previously known as GPR30), which is situated on both the cell membrane and intracellular membranes [10]. These receptors mediate the hormonal actions of E2, initiating a range of physiological responses.

The G-protein-coupled estrogen receptor (GPER) is ubiquitously expressed across a diverse array of tissues including, but not limited to, the central and peripheral nervous systems, placenta, lung, liver, prostate, ovary, mammary glands, kidneys, pancreatic islets, adipose tissue, cardiovascular system, musculature, skeletal system, and immune cells. Notably, the expression of GPER varies according to age, species, sex, and specific tissue type [10]. Estrogen receptors, ER-α and ER-β, exhibit an uneven distribution across various cells and organs, with certain tissues showing a predilection for higher levels of expression. For instance, ER-α is predominantly observed in the uterus, mammary glands, ovarian cells, prostate stroma, testes, epididymis in males, kidneys, bones, adrenal glands, and liver. Conversely, ER-β is principally located in the prostate epithelium, ovarian granulosa cells, colon, bladder, lung, salivary gland, and bone marrow, as referenced in [11–13].

**FIGURE 1:**
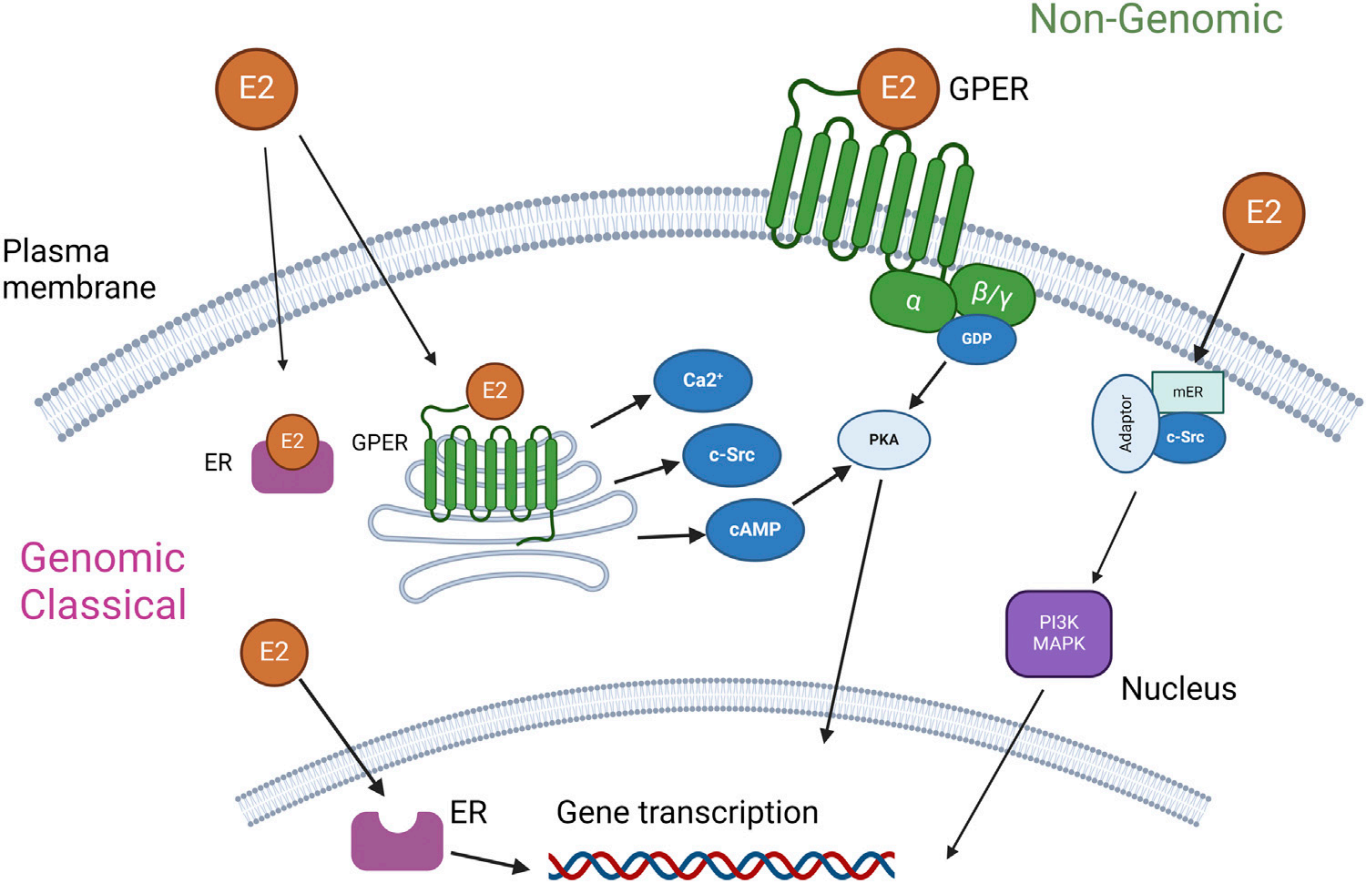
Estrogen receptors α, β, and GPER in the cell and their activation pathways [10, 14].

Figure 1 delineates the dual pathways of E2 activation, classified broadly into genomic and nongenomic categories based on the locus of activation. The classical genomic action of estrogen receptors initiates when ER in the cytoplasm binds to E2. This hormone-receptor complex then relocates to the nucleus, where it interacts with the estrogen response element within the DNA sequence. This interaction modulates the transcription of certain genes, with the resultant physiological effects manifesting over a timespan of several hours, due to their reliance on new protein synthesis [15]. In contrast, the nongenomic actions of E2 are characterized by their rapid onset, occurring within seconds to minutes. In one scenario, E2 engagement with GPER leads to the activation of various intracellular signaling pathways, including but not limited to the mitogen-activated protein kinase (MAPK) family, phosphoinositide 3-kinase (PI3K), Src tyrosine kinase (Src), adenylyl cyclase, and calcium-calmodulin-dependent kinases, as well as the mobilization of intracellular Ca^2+^ stores [14, 16]. Additionally, estrogen receptors present on the plasma membrane can be activated, interacting with adaptor proteins and other signaling entities to mediate swift signaling pathways such as those involving PI3K–Akt and MAPK [15]. This complex mechanism underpins E2 potential use in the therapy of different diseases.

E2 has been known for its role in female health, particularly in diseases such as osteoporosis [17], cardiovascular disease [18], type 2 diabetes [18], endometriosis [19], and neurodegenerative diseases [20]. Furthermore, it is also being investigated for its potential in acute treatments targeting innate immunity, where it has shown benefits like reducing leukocyte adhesion and migration [21, 22]. Experimental evidence suggests that estrogen or estrogen receptor (ER) agonists may be beneficial in organ donation [23, 24] and trauma-hemorrhage outcomes [25]. Additionally, E2’s protective effects on the cardiovascular system, such as inducing vasodilation and reducing vasoconstriction in hypoxia [26], make it a promising therapeutic agent. It has also shown protective effects in ischemia/reperfusion [27, 28] and brain death injuries [23, 24]. E2’s anti-inflammatory effects, comparable to glucocorticoids [29], further emphasize its therapeutic potential. Thus, E2 shows a remarkable potential to address different diseases.

**FIGURE 2:**
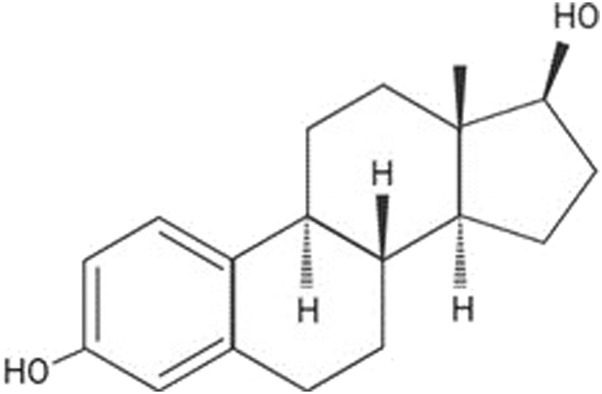
E2 2D molecular structure. Source: Jijana, 2023 [30].

Additionally, the E2 molecular structure is depicted in Figure 2, which illustrates its molecular mass of 272.4. While its solubility in water is limited to 3.90 mg/L at 27°C [31], E2 exhibits high solubility in organic solvents such as acetone, ethanol, dioxane [32], and its logP value of 4.01 indicates a high degree of lipophilicity.

Thus, there is an extensive therapeutic potential application for E2; in contrast, a successful drug delivery system can be a complex challenge, impacted by the E2 short half-life and rapid clearance, ending up with a narrow pharmacological window and with the need of larger dosage, and consequently, presenting higher toxicity and several adverse effects [33]. Nano-based drug delivery systems can overcome these challenges. This review aims to discuss the challenges and opportunities of lipid-based and polymeric nanostructured systems for improving the solubility and absorption of E2, with a focus on safety and efficacy.

## Nanotechnology-based E2 delivery

### Lipid nano-based E2 delivery systems

Lipid-based nanocarriers are influenced by the physicochemical properties of the drug, as well as by the composition and preparation techniques used. These carriers have been successfully developed and patented for the treatment of various diseases. The market for nano-based drug delivery is projected to reach $142.8 billion by [34]. According to [35], this drug delivery system is on the cusp of clinical translation. Given the hydrophobic nature of E2, lipid nanosystems show promise for its safe and effective delivery.

While these nanosystems may be suitable for the encapsulation of both hydrophilic and lipophilic drug substances and can circulate through the bloodstream effectively, they may also pose a risk of cytotoxicity due to non-specific cellular uptake [36].

Tables 1, 2 collectively present 13 studies on nano-based E2 delivery systems. Lipid-based and polymeric are the most commonly used nanoparticles in the medical field for drug delivery systems. Table 1 details lipid-based nanostructures for E2 delivery, whereas Table 2 focuses on polymeric nanoparticles. Among these studies, the routes of administration most frequently examined include oral and subcutaneous, each covered in two studies, as well as transdermal administration, also reported in two studies. Nasal and intravenous (IV) routes were each explored in one study. The administration routes were not specified in the remaining five studies. Specifically, Table 1 highlights five studies on lipid nano-based E2 delivery systems: two studies investigated liposomes (LP), which are bilayer vesicles composed of lipids and phospholipids; one study examined a lipid-ethanol-drug nanoparticle (LED) system; and two studies explored nanoemulsions (NE).

**TABLE 1:** Lipid-based nanocarriers for E2 delivery: route of administration, composition, physical -chemical characteristics, manufacturing process, *in vitro/in vivo* performance.

Type of nanoparticle	Route of administration	Composition	Particle size (nm)	PDI	ZP (mV)	EE/DL (% mm)	Preparation methods	*In vivo* and/or *in vitro* performance	References
LED	Transdermic	1,1 Di ((Z) - octadec-9-en-1-yl) piperidin-1-ium iodide	167.4	0.51	22.7 ± 6	74.3	modified ethanol injection method	LED - E2 permeated deeper and delivered to the dermal layer	Marepally S 2013
LP	-	DOPC DOPC: DDAB DOPC:DMPG	217 ± 6 199 ± 6 207 ± 2	0.124 ± 0.038 0.136 ± 0.006 0.113 ± 0.002	−0.46 ± 2.93 +26.72 ± 4.06 −28.00 ± 4.61	41.7 ± 4.2 51.2 ± 3.6 42.8 ± 2.9	thin-film hydration technique	DOPC liposomes with cationic DDAB had the highest E2 loading capacity and better cellular uptake	Bowey K 2014
LP	Subcutaneous	Main lipid (DPPC, DMPC or POPC), DDAB, cholesterol and E2 (molar ratio 1/0.2/0.6/0.2, total lipid concentration: 25 μM); HEPES buffer 10 mM (pH 7.4); PE-PEG2000 (2 mol%)	138 ± 4	0.06 ± 0.02	+27 ± 3	3.0 ± 0.9	thin-film hydration technique	E2 in LP can be a useful tool to indicate molecular mechanisms related to ERα	Gallez A 2020
NE	-	Flaxseed oil (w-3 polyunsaturated fatty acid a- linoleic acid), Lipoid^®^ E80 (2.4%, w/v), DSPE-PEG2000 (0.3%, w/v), deionized water, DOTAP (0.3%, w/v), chloroform, ethanol	138 ± 9	0.14	−33.80 ± 2.45	92.6 ± 4.6	microfluidization technique	E2 NE inhibited vascular smooth muscle cells (VSMC) and supported endothelial cells in connection with MAPK signaling	Deshpande D 2013
NE	Buccal Transmucosal	Transcutol P™, PEG 400, Tween 80, and deionized water	14.92	0.487	-	97.50	ultrasonication	A successful formulation was prepared, and it could be a promising administration route for E2 delivery	Abdella A 2022

Abbreviations: LED, Lipid - Ethanol - Drug Nanoparticle; LP, Liposome; NE, Nanoemulsion; LP, liposome; PDI, polydispersity index; ZP, zeta potential; EE, Encapsulation efficacy; DL, drug loading; DMPC, dimyristoyl phosphatidyl choline; DMPG, dimyristoyl phosphatidyl glycerol; DPPC, dipalmitoyl phosphatidyl choline; DOPE, dioleoyl phosphatidyl ethanolamine; DDAB, dimethyldioctadecylammonium; DSPC, 1,2-distearoyl-*sn*-glycero-3-phosphocholine; mPEG DSPE, 1,2-distearoyl-*sn*-glycero-3-phosphoethanolamine-N-[methoxy (polyethylene glycol)-2000; SPC, soybean phosphatidylcholine; mPEG2000-DSPE, methylpolyethyleneglycol-1, 2-distearyl-phosphatidylethanolamine conjugate; DMAP, p-dimethylamino pyridine; PRL, pH-responsive lipid derivative; HSPC, hydrogenated soybean phosphatidylcholine; DMPG-Na, 1,2-Dimyristoyl-*sn*-glycero-3-phosphoglycerol sodium salt; DSPG-Na, Distearoyl-*sn*-glycero-3- phosphoglycerol sodium salt; SA-3 M, 2,4,6-trimethoxybenzilidene-pentaerythritol coupled with Stearic acid; CTM-Ag, Clotrimazole-silver complex; PEG-660 stearate/Solutol HS15, 12-hydroxystearic acid-polyethylene glycol copolymer. 1,2-Dioleoyl-sn-glycero-3-phosphocholine (DOPC), 1,2-dioleoyl-sn-glycero-3-phosphoethanolamine (DOPE), and 1,2-distearoyl-sn-glycero-3-phosphoethanolamine- N- [maleimide (polyethylene glycol)-2000] (ammonium salt) (DSPE-PEG (2000) Maleimide), DPPC (palmitic acid; 16 atoms of carbon) and DMPC (myristic acid; 14 atoms of carbon) were selected as saturated lipids. 1-palmitoyl-2-oleoylsn- glycero-3-phosphocholine (POPC); (flaxseed oil) rich in the ω-3 polyunsaturated fatty acid (PUFA) α-linolenic acid; 1,2-dioleolyl-3-trimethylammonium-propane (DOTAP).

**TABLE 2:** E2 Polymeric nanoparticles: route of administration, composition, physical -chemical characteristics, manufacturing process, main results and target disease studied.

Type of nanoparticle	Route of administration	Composition	Particle size (nm)	PDI	ZP (mV)	EE/DL (% mm)	Preparation method	Main results	Target disease	Reference
NC	-	ABA triblock copolymer, (PEG–PBA–PEG) + olive oil	97 ± 22 to 384 ± 40	0.782 to 0.204	-	29.0 to 70.4	emulsification-solvent diffusion	It is possible to develop a NC using PEG block of copolymer as stabilizer, however, to reduce the size, it is necessary to use a minimal amount of surfactant	-	Khoee S, 2010
NS	Oral	PLGA	115.3 ± 2.5	-	+92.4 ± 3.2	51.2 ± 3.8	emulsion-diffusion-evaporation	E2-Nano particles better treated postmenopausal metabolic syndrome by prevention or reversion of the weight gain, dyslipidaemia and<!--Soft-enter Run-on-- > insulin resistance	CVD	Mittal G, 2009
NS	Transdermic	PLGA PLGA + PVA	110.0 ± 41.0 106 ± 30.9	-	-	0.93 ± 0.05 0.43 ± 0.03	antisolvent diffusion with preferential solvation	E2-loaded PLGA nanoparticles prepared by antisolvent diffusion method with preferential solvation confirmed sustained-release, with higher skin permeation and improved bone mineral density	Osteoporosis	Takeuchi I, 2017
NS	-	PLA: PEG - PLGA:PLA	49.4 ± 2.2	0. 194	−58.2 ± 3.0	-	water/oil emulsion and ultrasonication	Focusing on the development of the method, it allowed a better measurement of release rate for hydrophobic drugs than the conventional method	-	Gil D, 2018
NS	-	2% PLA–PEG–E2 93% PEG–diazobenzene–PLA	168 ± 3	0.13 ± 0.02	∼ zero	20	solvent exchange method	17β-E2-conjugated polymersomes targeted as polymeric hypoxia-responsive drug delivery nanocarriers bind to the ER on the surface of breast cancer cells and internalize them, releasing the active drug and reduce the cell viability	Breast cancer	Mamnoon B, 2020
NS	Subcutaneous	PLGA + PVA	256 ± 2.3	0.158 ± 0.02	∼ zero	-	single-emulsion technique and ultrasonication	PLGA-E2 nanoparticles heightened the stimulation of the uterus and showed cognitive beneficial effects	Spatial memory and Uterine stimulation (Hormone therapy)	Prakapenka AV, 2020
NS	Intravenous	PLGA	181 ± 1.2	0.236 ± 0.008	−4.18 ± 0.78	58.31 ± 9.17	emulsion solvent diffusion	Successfully developed a stable and biocompatible NP targeting bone to deliver E2	Osteoporosis	Guo Y, 2021
Gelatine	Nasal	WS -E2- GN 5% β-CD-GNP 10% β-CD-GNP	301.6 ± 74.7 315.7 ± 96.8 362.3 ± 151.4	-	+8.13 ± 4.80	94.6 ± 16.9 85.2 ± 4.9 95.5 ± 8.2	modified desolvation method	Gelatine nanoparticles successfully delivered E2 treatment of ischemic stroke	Neurologic disorders, stroke	Joachim E, 2020

Abbreviations: PDI, polydispersity index; ZP, zeta potential; EE, Encapsulation efficacy; DL, drug loading; PLGA, Poly (lactide-co-glycolide); PVA, polyvinyl alcohol; poly (ethylene glycol)–poly (butylene adipate)–poly (ethylene glycol); poly (lactic acid):polyethylene glycol (PLA:PEG) and 50:50 poly (lactic-co-glycolic acid):poly (lactic acid) (PLGA:PLA); Polylactic acid (PLA); 2-hydroxypropyl-β-cyclodextrin (β-CD).

Liposomes, which are phospholipid vesicles encapsulating a hydrophilic space with at least one lipid bilayer, boast the distinct advantage of being able to incorporate both lipophilic and hydrophilic compounds. This trait broadens the spectrum of deliverable drug substances. The flexibility and width of the liposome bilayer, which are crucial factors affecting E2 encapsulation, are influenced by bilayer composition, as elucidated by Gallez et al. (2020) [37]. Notably, the inclusion or exclusion of cholesterol impacts the physical state and structural dynamics of phospholipid chains; cholesterol serves to densify the lipid bilayer. Glycero-3-phosphocholine (POPC), containing an unsaturated cis-9 lipid chain (oleic acid), was selected for E2 encapsulation due to its enhanced flexibility. POPC also demonstrated superior loading capacities compared to 1,2-dimyristoyl-d54-sn-glycero-3-phosphocholine (DMPC) and 1,2-dipalmitoyl-sn-glycero-3-phosphocholine (DPPC). Furthermore, the saturation and length of the fatty acid chains are determinants of the lipid bilayer’s thickness and membrane robustness.

Innovative strategies utilizing novel materials for E2 lipid nanostructures have been shown to enhance therapeutic efficacy. Notably, LEDs have been developed with a new lipid class that includes heterocyclic head groups and oleyl chains, which promote superior transdermal penetration. Within this context, 1-Di-(Z)-octadec-9-en-1-yl) pyrrolidin-1-ium iodide (Cy5) demonstrated the most effective transdermal enhancement. E2 interaction with the bilayer is not limited to the phospholipid head but extends to the lipophilic core, primarily associated with the acyl chains of the lipid membrane. This interaction was exploited by Heger et al. (2015) [38] who replaced cholesterol in the hydrophilic part of the lipid bilayer with E2, preserving the hydrophilic properties while improving targeting to estrogen receptors (ERα and ERβ). E2 enhanced transfection efficiency by modulating plasma membrane properties and increasing liposome uptake into cells. The cationic nature of the liposomes further strengthened interactions with negatively charged cell membranes, enhancing the intracellular delivery of antisense oligonucleotides (ASOs). Additionally, E2 promoted the proliferation of estrogen receptor-positive cells, such as MCF-7, thereby increasing the overall therapeutic efficacy. This dual functionality of E2 improved both cell targeting and treatment efficiency.

Nanoemulsions (NEs) are oil-in-water nanocarriers that are heterogeneous and thermodynamically stable. These structures carry lipids that may function as surfactants, forming barriers that enhance the absorption of drug substances. NEs have been utilized to create promising mucoadhesive buccal films for E2 delivery, employing non-toxic and biocompatible components like PEG 400, polysorbate 80, and transcutol®P, which are commonly found in topical formulations. Additionally, ω-3 PUFA alpha-linolenic acid, a cardiovascular risk-lowering agent from flaxseed oil, was combined with E2 to form an NE with bioactive properties aimed at treating restenosis. Even in the absence of a drug substance, NEs demonstrated inherent bioactive effects, such as inhibiting cell proliferation. Egg lecithin was incorporated as a stabilizer, and DOTAP was used to impart a positive charge to enhance membrane transport. DSPE-PEG2000 played a role in prolonging the exposure of E2 to the vasculature by decelerating the clearance of the formulation.

The encapsulation of E2 within liposome formulations is characterized by a particle size range of 138–217 nm, a polydispersity index (PDI) spanning from 0.06 to 0.136, and a zeta potential fluctuating between −28.0 and +27.0 mV. The LED exhibits a mean particle size of 167.4 nm, a PDI of 0.51, and a zeta potential of 22.7 ± 6 mV. Additionally, two E2 NE have been reported with particle sizes ranging from 14.9 to 138 nm, PDIs between 0.14 and 0.487, and zeta potentials of −33.80 ± 2.45 mV.

The influence of particle size on the therapeutic efficacy of lipid nanoparticles is critical. Within the target tissue, the retention of lipid nanocarriers can be attributed to the size constraints imposed by capillary pores and the interstitial spaces. Optimal particle size for sustained-release antitumor drugs is typically within the range of 50–200 nm. In contrast, sizes starting from approximately 200 nm are indicated for intravenous (IV) or intramuscular (IM) administration. Lipid nanocarriers, such as liposomes, may exhibit prolonged tissue retention or phagocyte-mediated clearance when their size exceeds 100–150 nm after extravasation from the vasculature.

The PDI serves as an indicator of colloidal dispersion uniformity, with values approaching 0.01 signifying a uniform particle size distribution, and values near 1.0 indicating significant polydispersity. For lipid-based carriers, a PDI below 0.3 is generally accepted as indicative of a homogeneous particle population. Empirical data reveal a range of PDI from 0.06 ± 0.02 to 0.35 ± 0.04, although Marepally et al., 2013, reported a notably higher PDI of 0.51 for an E2 liposome formulation [39].

The zeta potential, an electrokinetic parameter, is used to assess the physical stability of nanoparticles, which includes their propensity for aggregation or agglomeration as a result of Ostwald ripening. Additionally, this parameter is influential in enhancing cellular uptake and determining the effectiveness of nanoparticle coatings. It is observed that lipid nanocarriers with positive zeta potentials ranged from 22.7 ± 6 to 27 ± 3 mV. The inclusion of a cationic surfactant such as DDAB can positively charge the nanocarrier, potentially increasing the encapsulation efficiency (EE) of negatively charged compounds like E2. Despite this, the highest EE of 97.50% was achieved using transcutol P^®^, a solubilizing agent commonly employed in drug formulations [39]. Its chemical structure, containing diethylene glycol monoethyl ether, enhances the solubility of poorly soluble drugs. The hydrophilic-lipophilic balance of transcutol P^®^ plays a pivotal role in drug solubilization and encapsulation. A favorable balance increases EE by maintaining the drug in solution during formulation, thereby improving its encapsulation within liposomes. In contrast, Gallez et al. (2020) demonstrated successful *in vivo* results with a low EE of 3.0%, highlighting that effective drug delivery to the target site is possible even with suboptimal encapsulation [37].

The preparation method significantly influences the properties of lipid nanoparticles. The thin-film hydration technique, employed in the preparation of E2 liposomes in two out of five studies, is a time-honored and straightforward approach for creating multilamellar vesicles. However, this method’s reliance on organic solvents presents a challenge for complete solvent removal, which can be problematic particularly for scalability and achieving high entrapment efficiency. Yaseen et al. (2021) [40] highlight the environmental concerns associated with non-green solvent use in pharmaceutical manufacturing, advocating for the adoption of sustainable solvents to mitigate pollution and reduce costs and environmental impact.

The modified ethanol injection method, as presented by Marepally et al. (2013) [39], involves the injection of an ethanol-phospholipid solution into an aqueous phase, with the ethanol volume being a critical factor for uniform vesicle formation. This technique is noted for its simplicity, reproducibility, and the preservation of lipid integrity.

Deshpande et al. (2013) [41] utilized the microfluidization technique, a high-pressure homogenization process that relies on particle interaction mechanisms such as collision, cavitation, shear, and turbulent forces to achieve particle size reduction. Its primary advantage lies in the method’s scalability, which is important for pharmaceutical industry applications.

Lastly, ultrasonication, employed by Abdella et al. (2022) [42], utilizes ultrasonic energy to disaggregate large particles into smaller or more uniform ones. While probe-type sonicators are more efficient than bath-type, the localized effect of emulsification near the waveguide radiator limits the method’s suitability for industrial-scale production due to non-uniform particle size distribution.

The composition of E2 lipid nanoparticles and the methodology for their preparation provide efficacious drug delivery systems. These systems elicited a therapeutic response in human vascular culture cells [41] and exhibited heightened permeation efficacy [39, 42].

LED-E2 demonstrated enhanced permeation, significantly improving the delivery of E2 to the dermal layer [39] (Table 1). The LED nanoparticles, particularly those using the cationic lipid 1,1-Di-[(Z)-octadec-9-en-1-yl] piperidin-1-ium iodide (Cy5), showed superior permeation due to their 5-membered amine heterocyclic ring, which is more effective than 6-membered rings. These Cy5 nanoparticles, sized between 150 and 200 nm, efficiently penetrate the skin barrier, leading to a 1.54 to 22-fold increase in drug retention in the dermis compared to conventional enhancers like oleic acid. The permeation mechanism involves ionic interactions, membrane destabilization, and increased skin hydration, promoting the penetration of encapsulated drugs.

The permeation rate of NE E2 incorporated within a mucoadhesive film reached 15% over 10 h, which is threefold greater than the permeation expected from oral tablets. However, these results, derived from an *ex vivo* model and a predictive model, still demand validation through *in vivo* studies to establish a proof of concept and to deepen our understanding [42].

In a related experiment, the impact of E2 liposomes was assessed using ovariectomized female rats as a model. The presence of E2 influenced several parameters in the uterus, which is known to respond to ERα-genomic/nuclear E2 dependent pathways. These parameters included the uterus’s wet weight, the proportion of Ki67-positive epithelial cells, and the height of the luminal epithelium, effects that were absent in groups not receiving the hormone. These findings suggest that E2 liposomes are capable of being internalized and releasing their contents, thereby inducing the activation of the ERα genomic/nuclear pathway in a manner comparable to free E2. To summarize, the liposomes were specifically effective in activating the genomic/nuclear pathway of ERα, indicating that LP-E2 could serve as a valuable tool in elucidating molecular mechanisms associated with ERα. Additionally, the liposomes appeared to modulate specific action mechanisms of pathways.

It is also imperative to ascertain the activation pathways of E2 receptors. Gallez et al. (2020) [37] highlighted that E2 encapsulated in liposomes failed to activate the α receptor found in the cell membrane. Consequently, the liposome-encapsulated E2 is internalized and activates the genomic pathway without initiating membrane-mediated steroid signaling. Overall, cationic LP showed the highest E2 loading capacity and better cellular uptake. LED formulation presents a higher permeation and delivers E2 to the dermal layer. Also, a buccal transmucosal formulation could be a promising administration route for E2 delivery. The LP-E2 is a promising tool to study the molecular mechanisms related to E2 receptors; NE – E2 inhibited VSMC and supported endothelial cells in connection with MAPK signaling.

Lipid-based nanocarriers, including liposomes, NE, and LED systems, present effective methods for delivering E2, each offering unique advantages based on their composition and manufacturing processes. Liposomes, with flexible bilayers like POPC, enhance drug loading and release by accommodating both hydrophilic and lipophilic substances. LED systems, especially those with cationic lipids like Cy5, provide improved transdermal delivery through better skin penetration and drug retention. Nanoemulsions used in buccal films achieve superior permeation compared to oral tablets, making them effective for mucosal delivery. The selection of lipids and manufacturing techniques, such as thin-film hydration or microfluidization, critically influences particle size, PDI, and zeta potential, which in turn affect the carriers’ performance. These lipid-based systems offer promising routes for patient-friendly E2 administration, including transdermal, buccal, and potentially intravenous, enhancing E2’s solubility, absorption, bioavailability, and controlled release for effective drug delivery.

### Polymeric nano-based E2 delivery systems

The utilization of polymeric nano-based drug delivery systems emerges as a compelling methodology for the sustained and controlled release of therapeutic agents. Advantages inherent to polymeric nanoparticles include biodegradability, biocompatibility, non-toxicity, and the capacity for surface modification. These nanoparticles are versatile in encapsulating a wide range of pharmaceuticals, encompassing both hydrophobic and hydrophilic drugs. However, their interaction with the reticuloendothelial system can precipitate rapid clearance from the bloodstream, potentially necessitating increased or more frequent dosing. Additionally, they may accrue within hepatic tissues, diminishing their efficacy in other organ systems [36].

In the realm of polymeric nanostructures, a dichotomy exists between nanocapsules (NCs) — reservoir systems — and nanospheres (NSs) — matrix systems — based on their respective formulations. NSs are characterized by a polymeric matrix that either entraps the therapeutic agent or adsorbs it on the surface [43]. In contrast, NCs consist of a drug solution encased within an oily core, enveloped by a polymeric shell that modulates drug release kinetics. NCs are predominantly synthesized via nanoprecipitation, a process entailing interfacial polymer deposition subsequent to the displacement of a semi-polar hydrophilic solvent. NSs, on the other hand, are fabricated through a series of processes including solvent evaporation, emulsification, solvent diffusion, reverse salting-out, and also nanoprecipitation. Both NSs and NCs can be formulated either by the polymerization of monomers or by the dispersion of pre-existing polymers or biopolymers, with the latter including naturally occurring, noncytotoxic, biocompatible, and biodegradable polymers such as chitosan and gelatin. These biopolymers are intrinsic to living tissues and may significantly enhance the absorption of drugs compared to their free, unencapsulated counterparts [43].

Table 2 delineates a comparative analysis of eight studies focusing on polymeric nano-based E2 delivery mechanisms. Within these studies, six pertain to nanosphere-based systems (NS), one utilizes a nanocapsule (NC) with an olive oil core, and the remaining three employ nanoparticles derived from biopolymers such as gelatin.

The analysis of Table 2 reveals that the majority of the reviewed studies (five out of eight) predominantly utilized poly (lactide-co-glycolide) (PLGA) as their primary polymeric compound in formulating nanoparticles, with polyethylene glycol (PEG), and poly (lactic acid) (PLA) following suit. Notably, PLGA and PLA are frequently selected polymers for nanoparticle synthesis, as evidenced by the literature [36, 43].

In a novel approach, a composite of PLA and PEG (specifically PLA17000-PEG2000) was employed to fabricate a distinct type of polymeric nanoparticle characterized by a bilayer membrane enveloping an aqueous core. E2 was effectively conjugated to the surface of these polymersomes [44].

Alternative polymers, including those that are synthesized or lesser known, have also been explored for nanoparticle preparation. For instance, the polymer PEG-PBA-PEG, in varying concentrations, has been demonstrated to modulate the release rate of E2 effectively. Moreover, this nanoparticle maintained stability with a minimal application of surfactant [45].

A singular study highlighted the use of a biopolymer, specifically gelatin nanoparticles (GNP), which encapsulated E2 utilizing the complexing properties of cyclodextrins (CD). This combination resulted in a GNP-CD system that exhibited a prolonged and sustained E2 release profile [46].

The particle sizes of these polymeric nanostructures ranged significantly, from 49.4 ± 2.2 nm to 418 ± 11 nm, a variation attributable to the differing compositions and methods of preparation. Notably, a larger particle size was observed with a polymer conjugate under hypoxic conditions (2% oxygen availability), resulting in particles measuring 695 ± 32 nm, whereas, under normoxic conditions, the size was reduced to approximately 168 nm [44].

Diverse techniques have been applied to measure particle size, with Prakapenka et al. (2020) [47] utilizing both transmission electron microscopy (TEM) and dynamic light scattering (DLS) for assessing size and hydrodynamic diameter, respectively. Each method possesses inherent advantages and limitations, but when used in conjunction, they provide a comprehensive understanding of particle dimensions. The FDA endorses such combined measurements for critical material attributes and emphasizes the importance of detailing the specifics of the analysis, including the distinction between hydrodynamic and projected radii, as well as comparing ensemble and single particle data [48].

Regarding PDI, values at or below 0.2 are generally deemed acceptable for polymeric nanoparticles [49]. The studies summarized in Table 2 largely conformed to this standard, with exceptions noted in the work of Guo et al. (2021) [50], which reported a PDI of 0.236 ± 0.008, and Khoee and Hossainzadeh (2010) [45], with a PDI over 0.204.

The zeta potential of the nanoparticles varied widely, from −58.2 ± 3.0 mV to +92.4 ± 3.2 mV, reflecting the diverse compositions (Table 2). Positive zeta potentials were specifically recorded for nanoparticles in the studies by Mittal et al. (2009) [51] and Joachim et al. (2020) [46], which were attributed to the incorporation of a quaternary ammonium salt, didodecyl dimethylammonium bromide (DMAB), as a stabilizing agent, and the use of type A gelatin, which is positively charged at physiological pH, respectively [46].

EE of the polymeric nanostructures was reported between 20% and 94.5% (Table 2). The highest EE was associated with nanoparticles having an olive oil core. It was observed that the ratio of polymer to olive oil critically influenced the EE, with a notable decrease in loading efficiency at a 1:2 ratio (53.94%), suggesting an optimal threshold for this proportion [45].

Remarkably, the highest EE was achieved by nanoparticles formulated with gelatin [46]. Additionally, Gil et al. (2018) [52] explored a novel assay for assessing the sustained release of active pharmaceutical ingredients, focusing on drug-albumin interactions. Nevertheless, this method has yet to be validated through *in vivo* testing.

The pursuit of polymeric nanostructures led to the execution of five studies employing various emulsion-based methodologies: emulsion-diffusion-evaporation [50, 51], emulsification-solvent diffusion [45], water/oil emulsion [52], and single-emulsion [47], the latter two of which were augmented with ultrasonication. Additionally, researchers utilized the solvent exchange method [45] and a modified desolvation technique [53], as delineated in Table 2.

A specific antisolvent diffusion approach, characterized by preferential solvation, yielded PLGA nanoparticles with protracted release profiles and enhanced dermal penetration, culminating in improved bone mineral density. These outcomes were notably superior to those attained through the conventional emulsification-solvent evaporation technique [53].

In the context of E2 nanoparticles, five *in vivo* and two *in vitro* assessments were conducted (Table 2). The application of E2 nanoparticles in rodent models with hyperlipidemia induced by a high-fat diet underscored the potential of these polymeric nanoparticles for oral administration. A comprehensive analysis of pharmacokinetic and pharmacodynamic parameters—including maximum plasma/serum concentration (Cmax), time to reach Cmax (Tmax), area under the plasma/serum concentration-time curve (AUC), absolute bioavailability, absorption rate constant (Ka), elimination rate constant (Kel), elimination half-life (T1/2), mean residence time, mean absorption time—revealed the efficacy of the treatment [54]. For instance, a dose of 500 μg/kg of E2 encapsulated in PLGA nanoparticles exhibited a Cmax of 476.02 pg/mL, Tmax of 18 h, T1/2 of 11.84 h, and AUC of 34,212.08 pgh/mL. In contrast, a drug suspension (DS) at an equivalent dosage demonstrated a Cmax of 757.03 pg/mL, Tmax of 4 h, T1/2 of 5.46 h, and AUC of 11,339.20 pgh/mL, respectively [51]. Consequently, the E2-nanoparticle formulation displayed a lower Cmax and a delayed Tmax relative to the DS, while delivering an approximately threefold higher drug concentration over time, as evidenced by the AUC, indicating a sustained release profile. Furthermore, when the same dosage was administered intravenously as a DS, the resultant AUC was 55,725.67 pg*h/mL with a T1/2 of 4.60 h, suggesting a more rapid elimination and greater bioavailability compared to oral administration of the E2-PLGA nanoparticles, thereby extending the potential dosing interval.

Additionally, intravenous administration of E2-PLGA nanoparticles was found to enhance bone density. In this study, ovariectomized female rats were employed as an animal model and treated over a course of 9 weeks. These findings corroborate the efficacy of E2-PLGA nanoparticles in the therapeutic management of osteoporosis in postmenopausal women, with specificity and targeting to bone tissues being potentiated by the co-administration of bisphosphonates or iron oxide [50].

Beyond oral and intravenous routes, the delivery of E2 polymeric nanoparticles was explored via intranasal, subcutaneous (SC), and transdermal pathways. Transdermal administration was specifically chosen to circumvent the first-pass metabolism associated with oral administration of E2 as an intervention for osteoporosis. Experimental models included excised rat abdominal skin for *ex vivo* analysis and female Sprague-Dawley rats for *in vivo* studies. The findings demonstrated that E2-PLGA nanoparticles, with a polyvinyl alcohol (PVA) coating, exhibited higher skin permeability compared to traditional nanoparticle formulations and enhanced bone mineral density [53].

For SC administration, a study on middle-aged ovariectomized female rats, which received weekly doses of E2-loaded PLGA nanoparticles, reported improved cognitive functions in learning and memory relative to the control group. Nevertheless, the therapy was also associated with notable adverse effects, such as increased uterine stimulation, when juxtaposed with the administration of free drugs [47].

The intranasal delivery of GNP conjugated with CD utilized a male C57BL/6 J mouse model. The outcomes indicated a reduction in the area of cerebral infarction, surpassing even that achieved with water-soluble E2 encapsulation, hence demonstrating promise for the treatment of ischemic stroke [46].

Polymeric nanoparticles are designed to provide controlled, sustained release, maintaining therapeutic levels over time and offering stable delivery across various routes. Their preparation involves well-established methods that may not require specialized equipment, though precise control of manufacturing processes is needed to achieve the desired performance. Despite the promising results from various studies, the progression toward a definitive proof of concept remains limited, highlighting the need for further *in vivo* research to validate and enhance their therapeutic applications.

## Discussion

In comparison between polymeric and lipid nanoparticles, both displays economically viable and potentially scalable preparation techniques. Moreover, both forms of nanoparticles afford greater bioavailability of E2 than free E2 and enable targeted delivery to specific membrane or intracellular compartments. Overall, lipid-based nanocarriers excel in targeted and topical E2 delivery, while polymeric nanoparticles are advantageous for controlled, long-term release. The selection of nanocarrier type and delivery method should be guided by the desired therapeutic outcomes and specific characteristics of E2 delivery.

The nanotechnology-based E2 delivery system presents substantial potential for advancing the management of osteoporosis, breast cancer, and an array of neurodegenerative and cardiovascular disorders, in addition to its use in hormone replacement therapy. By virtue of its engineered drug-release mechanisms, this system offers a safer and more efficacious alternative for sustained treatment regimens compared to conventional free-drug approaches. Furthermore, nanotechnology facilitates the mitigation of the deleterious effects typically associated with conventional E2 delivery methods, thereby enhancing therapeutic safety and patient adherence to treatment protocols. Notwithstanding, the progression to preclinical research represents a pivotal hurdle that must be overcome. Consequently, the forthcoming research initiatives should prioritize the refinement of experimental protocols involving the administration of nanostructured E2 in animal models, thereby catalyzing the transition towards human clinical trials and expediting the development of innovative therapeutic solutions. This review synthesizes the current challenges, progresses, and prospects pertaining to lipid and polymeric nanocarrier systems for E2 delivery, underscoring their potential to revolutionize the treatment landscape of different ailments.

## Glossary

E2: 17β-estradiol
β-CD: 2-hydroxypropyl-β-cyclodextrin
DOPC: 1,2-Dioleoyl-sn-glycero-3-phosphocholine
DOPE: 1,2-dioleoyl-sn-glycero-3-phosphoethanolamine
CTM-Ag: Clotrimazole-silver complex
DDAB: Dimethyldioctadecylammonium
DMAB: didodecyl dimethylammonium bromide
DMAP: p-dimethylamino pyridine
DMPC: dimyristoyl phosphatidyl choline
DMPG: dimyristoyl phosphatidyl glycerol
DMPG-Na: 1,2-Dimyristoyl-sn-glycero-3-phosphoglycerol sodium salt
DOPE: dioleoyl phosphatidyl ethanolamine
DOTAP: α-linolenic acid; 1,2-dioleolyl-3-trimethylammonium-propane
DPPC: dipalmitoyl phosphatidyl choline
DL: Drug loading
DSPC: 1,2-distearoyl-sn-glycero-3-phosphocholine, mPEG
DSPE: 1,2-distearoyl-sn-glycero-3-phosphoethanolamine-N-[methoxy (polyethylene glycol)-2000
DSPE-PEG (2000) Maleimide: 1,2-distearoyl-sn-glycero-3-phosphoethanolamine- N- [maleimide (polyethylene glycol)-2000] (ammonium salt)
DSPG-Na: Distearoyl-sn-glycero-3- phosphoglycerol sodium salt
EE: Encapsulation efficacy
eNOS: endothelial nitric oxide synthase
ER: Estrogen receptors
ET-1: endothelin-1
GPER/GPR30: G protein-coupled estrogen receptor
HER2: growth factor receptor 2
HSPC: Hydrogenated soybean phosphatidylcholine
IM: Intramuscular
IV: Intravenous
LED: Lipid - Ethanol - Drug Nanoparticle
LP: Liposome
MAPK: Mitogen-activated protein kinase family
mPEG2000-DSPE: methylpolyethyleneglycol-1,2-distearyl-phosphatidylethanolamine conjugate
NE: Nanoemulsion
NC: Nanocapsule
NO: Nitric oxide
NS: Nanosphere
PDI: Polydispersity index
PEG-660 stearate/Solutol HS15: 12-hydroxystearic acid-polyethylene glycol copolymer
PI3K: phosphoinositide 3-kinase
PLA: Polylactic acid
PLA-PBA-PEG: poly (ethylene glycol)–poly (butylene adipate)–poly (ethylene glycol)
PLGA: Poly (lactide-co-glycolide)
PLGA:PEG: poly (lactic acid):polyethylene glycol
PLGA:PLA 50: 50 poly (lactic-co-glycolic acid):poly (lactic acid)
POPC: 1-palmitoyl-2-oleoylsn- glycero-3-phosphocholine
PRL: pH-responsive lipid derivative
PUFA: (flaxseed oil) rich in the ω-3 polyunsaturated fatty acid
PVA: polyvinyl alcohol
SA-3 M: 2,4,6-trimethoxybenzilidene-pentaerythritol coupled with Stearic acid
SPC: Soybean phosphatidylcholine
Src: Tyrosine kinase
ZP: zeta potential
